# Studying the Developing Brain in Real-World Contexts: Moving From Castles in the Air to Castles on the Ground

**DOI:** 10.3389/fnint.2022.896919

**Published:** 2022-07-13

**Authors:** Sam V. Wass, Louise Goupil

**Affiliations:** ^1^Department of Psychology, University of East London, London, United Kingdom; ^2^LPNC, Université Grenoble Alpes/CNRS, Grenoble, France

**Keywords:** neuroimaging, development, entrainment, environment, real-world, naturalistic, hyperscanning

## Abstract

Most current research in cognitive neuroscience uses standardized non-ecological experiments to study the developing brain. But these approaches do a poor job of mimicking the real-world, and thus can only provide a distorted picture of how cognitive operations and brain development unfold outside of the lab. Here we consider future research avenues which may lead to a better appreciation of how developing brains dynamically interact with a complex real-world environment, and how cognition develops over time. We raise several problems faced by current mainstream methods in the field, before briefly reviewing novel promising approaches that alleviate some of these issues. First, we consider research that examines perception by measuring entrainment between brain activity and temporal patterns in naturalistic stimuli. Second, we consider research that examines our ability to parse our continuous experience into discrete events, and how this ability develops over time. Third, we consider the role of children as active agents in selecting what they sample from the environment from one moment to the next. Fourth, we consider new approaches that measure how mutual influences between children and others are instantiated in suprapersonal brain networks. Finally, we discuss how we may reduce adult biases when designing developmental studies. Together, these approaches have great potential to further our understanding of how the developing brain learns to process information, and to control complex real-world behaviors.

“Problem-level assumptions set the course for the entire research program” (Edelman, 2016).
“There is more pleasure to building castles in the air than on the ground” (Gibbon, 1788).

## Introduction – The Problem: Most Current Approaches to Studying Brain Development in Human Infants Are Reductionist

When psychologists and (more recently) cognitive neuroscientists want to study a particular real-world cognitive operation (such as inhibition), they usually do this by building an experimental task (such as the Stroop task) that aims to mimic the real-world cognitive operation in a controlled manner, away from individual instances and individual settings (Danziger, 1994; Hatfield, 2002). From the start, we have been aware that this approach has limitations (Bronfenbrenner, 1977; Aanstoos, 1991; Anderson et al., 1999; Shamay-Tsoory and Mendelsohn, 2019; Sonkusare et al., 2019) [although see Holleman et al. (2020)]. But it is always worth reminding ourselves what some of these limitations are—particularly when it comes to studying brain function *in vivo*, and during development. In the animal (Miller et al., 2022) and adult (Newen et al., 2018) literatures, important advances have been made to develop more ecological paradigms. However, in the developmental literature, most studies still suffer from at least two important limitations:

The first problem is that these experimental tasks often differ in a number of ways from the specific real-world cognitive operation that they are intended to mimic (Shamay-Tsoory and Mendelsohn, 2019). For example, most of our knowledge of how our brains learn to process social information comes from tasks that record visual event-related potentials while presenting pictures containing different types of social information to children (de Haan et al., 2013; Grossmann, 2015). But these visual event-related potentials are always measured relative to moments where the pictures suddenly appear and disappear, or relative to the repeated presentation of exact sequences of events that reoccur. In the real world, though, individual pictures rarely if ever flash on and off out of the darkness, and specific sequences of events virtually never reoccur. Similarly, in studies examining language acquisition, auditory evoked potentials tend to be measured relative to the presentation of individual nouns or sentences (Junge et al., 2021), whereas real-world language comprehension critically requires parsing single words out of a complex, dynamic speech stream.

Another near-universal factor in experimental studies is that both the events themselves, and their exact timings, tend to be decided by the experimenter, and not the participant. In the real world, though, behavior does not happen just through passive, serial-order responses to external stimuli (Phillips, 1971; Marr, 1985; Smith and Gasser, 2005; Spivey and Dale, 2006; Yu and Smith, 2013; Kolodny and Edelman, 2015; Edelman, 2016). As Dewey first noted over hundred years ago “[w]hat we have is a circuit, not an arc or broken segment of a circle. […] The motor response determines the stimulus, just as truly as sensory stimulus determines movement” (Dewey, 1896) (p.365). This point applies to my interactions with my physical environment: how I move and where I attend influences what information I receive. And it also applies to my interactions with my social environment: how I behave toward others influences what I receive from them in return.

In the lab, researchers present children with standardized and specifically designed stimuli whose specific features have often been chosen based on their own (adult, expert in child development) understanding of what is relevant and important for children. These assumptions may sometimes match caregivers’ assumptions, and thus partially match what infants are given perceptual access to in their daily lives. But they can also critically differ, and a better approach might be to let the data decide what aspects of the naturalistic environment are most developmentally relevant to infants, and children.

The second general problem is that these approaches rely on the assumption that cognitive operations *can* be abstracted and encapsulated by stable and context independent mental state concepts such as “attention” or “cognitive control” (Pessoa et al., 2022)—i.e., that, for example, cognitive control measured at one time and using one paradigm relates meaningfully to cognitive control measured using a different type of paradigm, and cognitive control as it is deployed in ecological contexts (Campbell, 1957; Neisser, 1977; Broadbent, 1993; Kingstone et al., 2003, 2008; Doebel, 2020). In fact, it is rarely, if ever, tested whether experimental simulacra do actually mimic the real-world cognitive operation that they were designed to imitate (e.g., whether people who perform better at an experimental simulation of inhibition actually show better inhibition in real-world settings) (Sonkusare et al., 2019). What research there is suggests that both individual differences (Neisser, 1977; Awh et al., 2007) and transfer effects following cognitive training (Holmes et al., 2019) are in fact remarkably specific to minor details of the experimental paradigm used.

Indeed, such abstract concepts may rarely—if ever—strictly correspond to distinct neural structures (e.g., there is no strict boundary at the neural level between “emotions” and “cognition”) (Pessoa et al., 2022); rather, neural architectures appear to be geared toward solving specific problems that depend on the characteristics of the world that cognitive agents live and develop in Pessoa et al. (2022). In other words, although mental state concepts constitute useful shortcuts to talk about cognitive operations, understanding how neural systems support behavior requires research that documents how cognitive agents solve specific real-world problems. Relatedly, in the adult literature, authors have advocated for 4E approaches, which propose that experimental paradigms should always attempt to reflect the fact that cognition is necessarily embodied, embedded, extended and enacted (Newen et al., 2018).

In the following, we review recent developments in how we study brain function across adult and developmental cognitive neuroscience, and we discuss possible new future research directions that we hope will in future allow us to alleviate some of these problems, and to move closer to understanding how human children develop through repeated interactions with their environments.

Our discussion is in five sections (see Table 1). In Part 1, we examine studies that have developed new approaches to measure how complex, naturalistic stimuli are perceived. In Part 2, we consider research which examines our ability to parse our continuous experience into discrete events, and how this ability develops over time. In Part 3, we consider the role of children as active agents in selecting what they sample from the environment one moment to the next. In Part 4 we consider new approaches that measure how mutual influences between children and others are instantiated in the brain. Finally, in Part 5, we discuss research strategies that may enable us to limit the influence of experimenters’ own beliefs when designing experiments, which appears especially important given the discussion developed in the preceding sections.

**TABLE 1:** A summary of the main problems, and solutions, discussed in this paper.

Problem	Solution	Examples of papers using that solution
Problem 1: Many approaches to studying brain function rely on measuring brain changes to aspects of the stimulus (such as appearances, disappearances and repetitions) that rarely if ever occur in real-world settings.	New approaches allow us to study entrainment between a brain and complex, continuous stimuli as it encounters them in everyday settings.	Kalashnikova et al., 2018; Jessen et al., 2019, 2021; Attaheri et al., 2021; Rocha et al., 2021; Menn et al., 2022
Problem 2: Event-boundaries, and the structures of specific experimental events, are generally defined *a priori* by the experimenter, rather than “on the fly” by the agent embodied in their environment.	Analyze the temporal interdependencies between real-world event sequences generated by children and their social partners, and measure how the predictability of an event sequence relates to the predictability of brain activity patterns.	Zacks et al., 2001; Kurby and Zacks, 2008; Simony et al., 2016; Monroy et al., 2019; Zacks, 2020; Ghilardi et al., 2022
Problem 3: children actively sample their environment. Behavior does not happen just through passive, serial-order responses to external stimuli; rather, the response determines the stimulus just a truly as vice versa.	Measure bidirectional inter-relationships between fluctuating brain states and real-world behaviors.	Robertson et al., 2012; Hellyer et al., 2014; Fagerholm et al., 2015; Lynn et al., 2021; Plenz et al., 2021
Problem 4: attentional and affective states are shared between children and other people, and children and their caregivers mutually affect one another’s actions and perceptions during social interaction	Record from two interacting brains during real-world naturalistic interactions.	Hoehl and Markova, 2018; Markova et al., 2019; Wass et al., 2020; Turk et al., 2022
Problem 5: experimenters decide *a priori* what aspects of development are most important to study in children.	Use reverse-correlation approaches to present naturally or pseudo-naturally occurring variations and use participants’ responses to reconstruct the mental models that drove their judgments in a data-driven, rather than experimenter-driven, fashion. Design and interpret experimental studies on the basis of corpus analyses that help describe what infants’ actual inputs are. Rely on diverse teams of researchers to design studies.	Jack et al., 2012; Richardson et al., 2018; Burred et al., 2019; Kamps et al., 2021; Urassa et al., 2021

## Section 1 – The Passive Perception of Complex, Naturalistic Stimuli

In the real world we virtually never encounter a stimulus that flashes on and off, in isolation, out of the black—despite the popularity of this type of stimulus in neuroimaging studies (Shamay-Tsoory and Mendelsohn, 2019). Rather, the real world is a complex, dense, continuous mismash of electromagnetic information, through which our sensory systems have developed to navigate. Reflecting this, an increasing number of studies have started to measure brain responses during the passive perception of complex, naturalistic stimuli that approach the complexity of real-world stimuli. Practically, these normally take the form of audio and video recordings that are presented identically to multiple participants.

A large body of research has looked at how temporal activation patterns in our brain respond to periodic and aperiodic temporal structures in our everyday environments (Haegens and Golumbic, 2018; Rimmele et al., 2018; Lakatos et al., 2019). Of this, the largest body of evidence looks at temporal structures in everyday natural speech (Giraud and Poeppel, 2012; Doelling et al., 2019; Poeppel and Assaneo, 2020). Several recent papers have used EEG and fNIRS to demonstrate that infants show dynamic neural tracking to visual information (Jessen et al., 2019) and natural speech (Liu et al., 2017; Kalashnikova et al., 2018; Jessen et al., 2019; Attaheri et al., 2021; Barajas et al., 2021; Menn et al., 2022) in pre-recorded videos. These studies have mainly used variants of the Temporal Response Function (Jessen et al., 2021), which essentially regresses the stimulus (e.g., the auditory envelope of speech) onto the neural activity (or vice-versa).

It remains to be seen, though, how mechanistically the developing brain processes the environment. A particularly important question is whether dynamic stimulus processing is driven by oscillatory entrainment (endogenous oscillatory activity in the brain becoming coupled with oscillatory activity in the stimulus) or by contingent responding (the brain showing an evoked response whenever a stimulus occurs) during early development (Wass et al., 2021a). It also remains to be seen, for example, how endogenous attention, and the comprehensibility of this stimulus, affect neural tracking during early development (see van der Ghinst et al., 2019; Barajas et al., 2021).

## Section 2 – Learning to Parse Our Continuous Experience of the Real-World Into Discrete Events

Our experience of the real world is dynamic and continuous. But when we are paying attention to real-world events, Event Segmentation Theory (EST) (Zacks et al., 2001, 2010; Kurby and Zacks, 2008; Zacks, 2020) states that we segment events hierarchically on a coarse-fine spectrum so as to make prediction of the near future easier; we use “event models” stored in working memory to match expectations with what we are currently observing, and we update these models at event boundaries when such a model is no longer accurately predicting what we see. Evidence of maintaining event models comes from research demonstrating that we can predict what happens before an event boundary with ease but have difficulty predicting what happens after the boundary (Zacks, 2020). EST gives a parsimonious account of what mental representations underlie sustained attention. In traditional views of sustained attention, working memory (WM) is thought to be key for holding task-relevant information in mind.

For young infants, WM capacity is thought to be low (Colombo and Cheatham, 2006). Based on Event Segmentation Theory, therefore, we might infer that infants lack any hierarchical structure to their behavior; their exploration of new objects might be fragmented, and might not be segmented into discrete events embedded within overarching hierarchical structures. Intuitively, this prediction seems correct but it has not, to our knowledge, been tested. To do this, we could analyze the temporal interdependencies between real-world event sequences generated by children, and measure how the predictability of an event sequence relates to the predictability of brain activity patterns. We could also examine how the predictability of external events (e.g., the movements and gestures that a parent makes while playing with a child) relates to the predictability of brain activity. Here, we predict that that degree of entrainment shown by the child to the hierarchical structures of events might increase over time. Again, though, this prediction is untested.

Another open question is: *how* does the ability to parse continuous experience into discrete, meaningful events develop over time? Even during early infancy (3-months-old in linguistic studies), statistical learning of co-occurrences can guide our predictions about what we are seeing and hearing (Saffran, 2003; Baldwin et al., 2008; Stahl and Feigenson, 2015; Saffran and Kirkham, 2018). There is evidence that word learning—which requires singling out specific words and objects and matching them—is supported by the cross-situational statistics that learners can draw from multiple encounters with word-object associations across varying contexts (Smith and Yu, 2008; Bergelson and Aslin, 2017). Similarly, it has been suggested that statistical learning abilities may enable the child to parse their continuous everyday experience into meaningful event subunits (Levine et al., 2017; Conway, 2020). However, this idea also remains currently untested.

## Section 3 – Children Actively Sampling From the Environment

The research discussed above looks at how we passively process sensory information. But, as we discussed above, children continuously interact with their proximate environment. Behavior does not happen just through passive, serial-order responses to external stimuli; rather, the response determines the stimulus just a truly as *vice versa* (Dewey, 1896; Phillips, 1971).

In this section we consider: what can neuroscience tell us about how we dynamically control our behavior, moment-by-moment, “on the fly”? This question builds on research that looks at early foraging behaviors, in humans and animals. For example, modeling work has shown that just two parameters—stochastic gaze shifts and hysteresis (the intrinsic “stickiness” of attention states)—can accurately model gaze behaviors in younger (1-month-old) infants, but are less accurate for older (3-month-old) infants (Robertson, 2004, 2014). Similarly, attention allocation fluctuates more periodically over time during early compared with later infancy (Feldman and Mayes, 1999). One interpretation of these findings is that early orienting behaviors are relatively more determined by factors internal to the infant in a bottom-up fashion during early life; during later infancy, orienting behaviors start to become more influenced by the external properties of the environment in which the infant is located (Posner et al., 2014).

Recent research with adults has examined entropy production in the human brain, by quantifying detailed balance—i.e., the balance of likelihood between one possible transition (state A - > state B) and the opposite transition (B- > A) (Lynn et al., 2021). Adult brains nearly obey detailed balance at rest (Lynn et al., 2021). Given the intrinsic instability of younger brains, it seems plausible to predict that resting state entropy in younger brains ought to be lower, and that detailed balance is less likely to be maintained; however, this prediction is untested.

Research with adults has also examined the differences in the energetic state of the brain between a resting and an attentive state. Generally, the resting state is associated with near-critical dynamics, in which a high dynamic range and a large repertoire of brain states may be advantageous; whereas, a task-active (attentive) state induces subcritical dynamics, which is associated with a lower dynamic range, which in turn may reduce elements of interference affecting task performance (Hellyer et al., 2014; Fagerholm et al., 2015; Lynn et al., 2021; Plenz et al., 2021). According to the free energy minimization principle, biological systems must resist the second law of thermodynamics (i.e., a tendency to disorder), so that they do not decay to equilibrium (Friston and Stephan, 2007; Friston, 2010); one mechanism that they might use to do this is through sampling the environment, to actively minimize the surprise of each successive sensory sample (Sengupta et al., 2016; Schwartenbeck et al., 2019). Behavioral evidence in adults (Oudeyer et al., 2016; Ten et al., 2021) and children (Kidd et al., 2012; Begus and Southgate, 2018; Poli et al., 2020) has shown that attentional allocation and information seeking reflect how predictable and informative stimuli are to them. In adults, we know that neural representations of subjective confidence and surprise are related to information-seeking (Ligneul et al., 2018; Desender et al., 2019). But whether similar neural representations guide infants’ attention allocation and exploration remains unclear (though see Meyer et al., 2022). To test this, we could measure the ongoing, bidirectional inter-relationships between fluctuating brain states and real-world behaviors. One further prediction—which again is untested—is that the degree of energetic change (quantified as criticality) between a resting and attentive state should increase over developmental time.

## Section 4 – Examining Mutual Influences Between Children and Others

What infants perceive is not only determined by how they actively sample the world, but also by their caregiver’s decisions and actions. Babies spend most of their awake time with other people—e.g., caregivers. It seems critical, then, to understand how caregivers and infants *together* shape infant’s sensory inputs (Vygotsky et al., 1994; Feldman, 2007). This is not a unidirectional process, as caregivers’ actions are also largely dependent on their infants’ behavior, so it is essential to understand how attentional and affective states are shared between children and other people, and how children and their caregivers mutually affect one another’s actions and perceptions during social interaction.

Extensive behavioral research has been dedicated to this issue (Jaffe et al., 2001; Feldman, 2007; Yu and Smith, 2016; Wass et al., 2018), but only recently has there been an equivalent shift away from studying how our brains process a one-way flow of information—e.g., from senders to receivers—toward approaches that examine bidirectional information exchanges between multiple brains during social interaction (Risko et al., 2016; Redcay and Schilbach, 2019; Osborne-Crowley, 2020; Wass et al., 2020; Fan et al., 2021; Holroyd, 2022; Turk et al., 2022). In the developmental literature, a growing number of studies have started to investigate interpersonal brain couplings (IBC) in interacting adult-child dyads (Hoehl and Markova, 2018; Markova et al., 2019; Piazza et al., 2020; Wass et al., 2020; Turk et al., 2022).

Unsurprisingly, given the recentness of this work, and the wide-ranging and fundamental differences between the paradigms and analyses used in two-brain neuroimaging recordings compared with traditional one-brain recordings, there are currently numerous fundamental disagreements between researchers in how paradigms should be designed, and what measures and analyses should be used (Hamilton, 2021; Holroyd, 2022). An important step forward will be to better understand how coupled brain states relate to behavioral and physiological coupling. IBC is typically measured in situations where partners also have a common perceptual access to a shared environment (Hamilton, 2021), but without parsing out the contribution of the shared environment on each partners’ neural activity it remains difficult to interpret IBC’s functional significance (Holroyd, 2022). Moving in this direction probably requires event-locked approaches whereby IBC is examined with respect to specific “edges” that naturally occur during social interactions—such as the occurrence of mutual gaze, parental emphasis during speech, bursts of infant vocalizations, gaze shifts toward a joint focus, etc., (Haresign et al., 2022). Alternatively, joint measurements of behavioral, physiological and neural coupling and multiple regression approaches can also shed light on this issue (Nguyen et al., 2020; Piazza et al., 2020; Reindl et al., 2022). This will be important to reach a mechanistic understanding of how individual brains support collective behaviors such as joint attention and joint action that are thought to be crucial for early learning.

## Section 5 – Picking One Stream From Many: Experimenter’s Lenses and Distorted Pictures

So far, we have considered how our brain responds to isolated streams of sensory information in our environment (section 1) and how the ability to parse continuous streams of sensory information into discrete events may develop over time (section 2). We then discussed how, from early on in development, children’s experiences are largely determined by how they actively sample their environment (section 3), and how others respond to them (section 4). Studying these four elements is crucial because, in the real world, we are bombarded by a polyphony of different types of dynamic information from different sources. When experimenters investigate child development in the lab, however, they typically decide to present specific stimulus in a specific context and order. And even when they decide to observe children’s environment, they use a specific lens to analyze their data, and select specific variables of interest. In other words, in most cases, the decision about what to look at is decided *a priori* by the experimenter.

Recent research on language acquisition illustrates the type of problems that this can raise: while initial investigations suggested that children from low-socioeconomic status (SES) households hear far less words than children from higher SES households [the infamous ‘30-million word gap’, (Hart and Risley, 1995; Huttenlocher et al., 2007; Hirsh-Pasek et al., 2015)], later work suggested that this picture was in fact partially due to the experimenters’ decisions about what to look for—for example, by focusing on words that are directed to the child by the primary caregiver, rather than extending the word count to bystanders (Sperry et al., 2019; Dailey and Bergelson, 2021). Other researchers have made the comparable point that Western researchers may overemphasize the role eye contact in early social development—which is more important in Western parent-child interactions (where babies spend more time seated, and face-to-face) than in other cultures (where infants often spend more time carried, and facing in the same direction as their parents) (Feldman et al., 2006; Akhtar and Gernsbacher, 2008). Clearly, there is a similar risk in designing developmental neuroscience studies that the experimenter’s adult preconceptions may influence design and analysis decisions about what aspects of the naturalistic environment are most developmentally relevant to infants, and children.

One way to circumvent this is to look at how different types of inputs (e.g., linguistic, musical, visual etc.) are actually distributed in diverse naturalistic data (VanDam et al., 2016; Bergelson and Aslin, 2017; Clerkin et al., 2017; Mendoza and Fausey, 2021; Yu et al., 2021), and to use this information to design experimental paradigms, and interpret data collected in the lab. Recently, the use of day-long recordings has been democratized, which allows describing how infants’ linguistic (VanDam et al., 2016) or musical (Mendoza and Fausey, 2021) inputs are distributed in their daily environment. This also allows linking infant’s behavior measured in the lab with properties of their environment (e.g., Bergelson and Aslin, 2017), providing an insight into how daily life experiences shape the development of specific skills. As of yet, this has not been linked to brain development, but recent studies have shown how infant’s physiological responses vary throughout the day (Wass et al., 2019), and how infants’ environment impacts these responses (Wass et al., 2021b).

Data-driven approaches have also been developed to mitigate some of these problems. Instead of positing specific categories beforehand—which can create confirmation biases and demand effects—reverse-correlation approaches rely on the presentation of several naturally or pseudo-naturally occurring variations (Jack et al., 2012; Burred et al., 2019). Participants’ categorical or dimensional responses to this large corpus are then used to reconstruct the mental models that drove their judgments in a data-driven, rather than experimenter-driven, fashion. These methods have recently started to be applied to developmental neuroscience, where they allow researchers to measure children’s neural responses to stimulus categories determined in a data-driven, rather than experimenter-driven way (Richardson et al., 2018; Kamps et al., 2021).

Finally, problems related to experimenters’ culturally situated lenses can be partially alleviated by conducting cross-linguistic and cross-cultural studies. This approach is increasingly popular in adult research (e.g., Henrich et al., 2010), but it is important for developmental neuroscience and psychology, too—as the example related to linguistic input described above illustrates. Arguably, diversity in recruiting lab-members (Urassa et al., 2021) also has the potential to lead to more neutral experimental designs, because the relativity of each experimenter’s culturally situated beliefs should become obvious when they exchange ideas to design their studies.

## Conclusion

Since the very earliest days of psychology and cognitive neuroscience, we have been aware that using non-ecological experimental tasks to mimic real-world cognitive operations is an approach with intrinsic limitations. Recently, a number of approaches have been developed that offer new perspectives on understanding how our brains develop through our everyday, moment-by-moment interactions with our environments around us. These new approaches hold, we consider, great promise. In future, new approaches will allow better to understand how our brains process complex, often unpredictable, real-world sequences (sections 1 and 2). They will also allow us to better study children as dynamic, embodied, interactive agents who choose what they sample from their physical and social environment from one moment to the next (sections 3 and 4). Finally, they will allow us to move beyond basing our decisions as to what is important to study in child development purely on our own preconceived adult ideas (section 5).

We started this article with a quotation from Edward Gibbon, suggesting that using non-ecological neuroimaging paradigms to study purely internal mental constructs is like “building castles in the sky.” Taken together, the various approaches discussed in this paper have to potential to allow us to reach a better understanding of how human minds develop by learning to select information from complex and continuously evolving streams of information in the real-world environment. As Henry David Thoreau put it: “If you have built castles in the air, your work need not be lost; there is where they should be. Now put foundations under them” (Thoreau, 1854).

## Author Contributions

Both authors listed have made a substantial, direct, and intellectual contribution to the work, and approved it for publication.

## Conflict of Interest

The authors declare that the research was conducted in the absence of any commercial or financial relationships that could be construed as a potential conflict of interest.

## Publisher’s Note

All claims expressed in this article are solely those of the authors and do not necessarily represent those of their affiliated organizations, or those of the publisher, the editors and the reviewers. Any product that may be evaluated in this article, or claim that may be made by its manufacturer, is not guaranteed or endorsed by the publisher.
